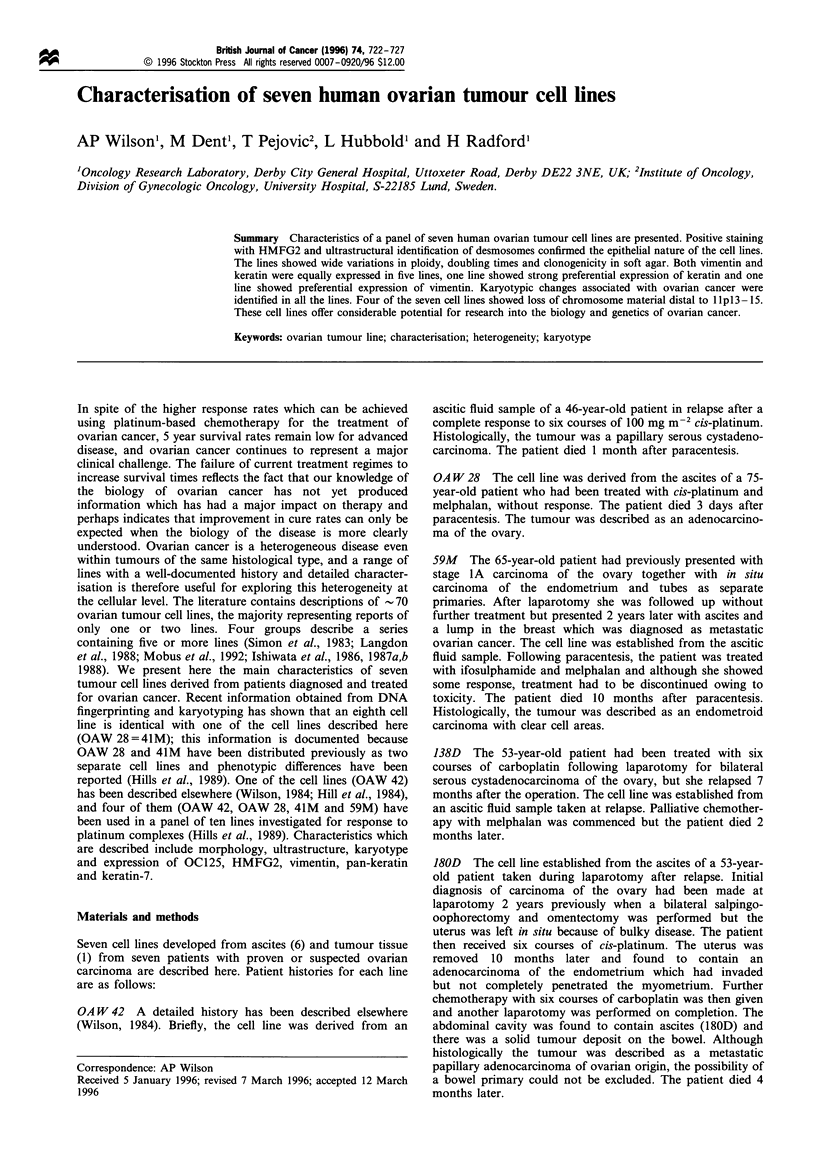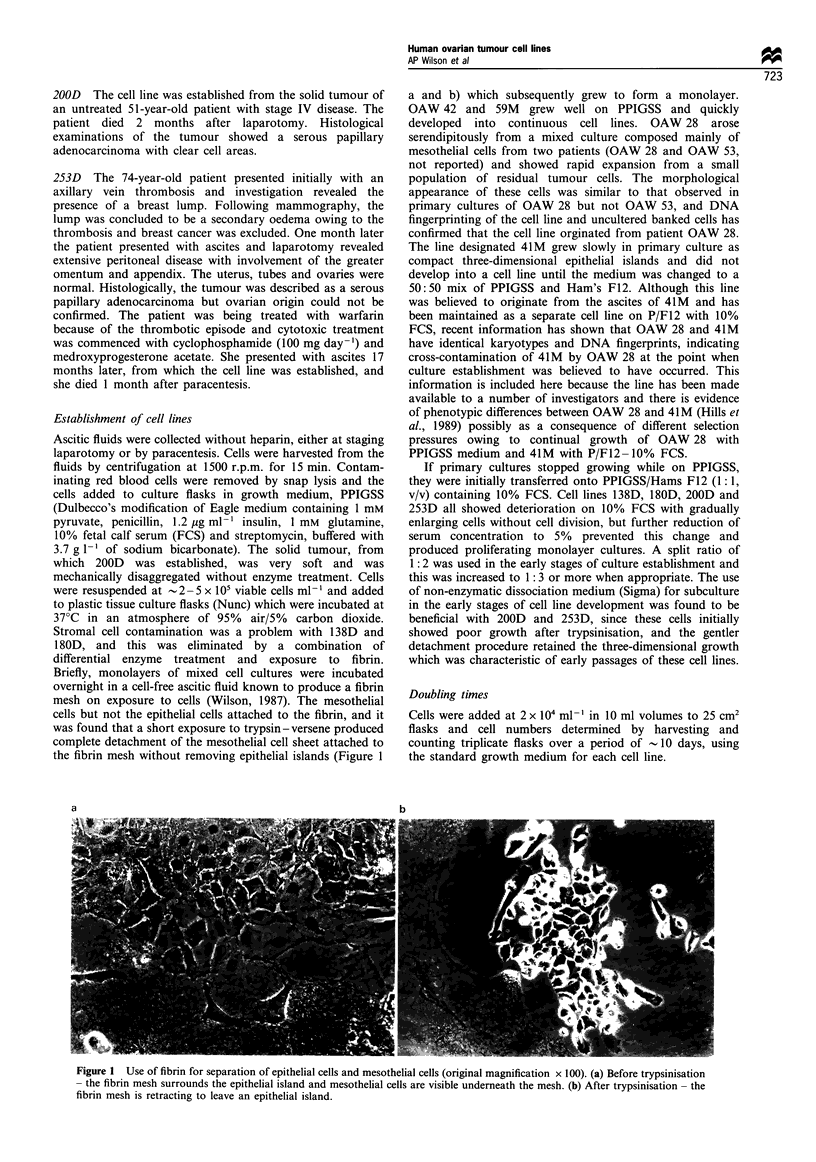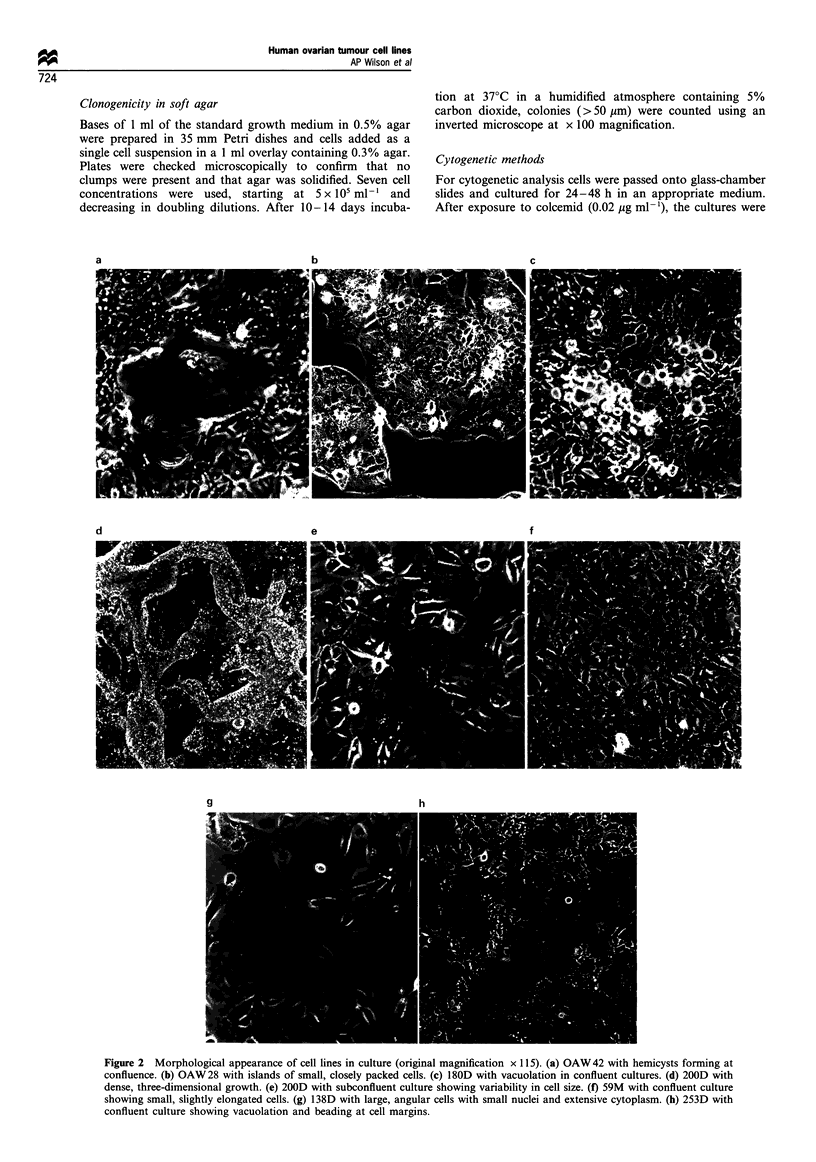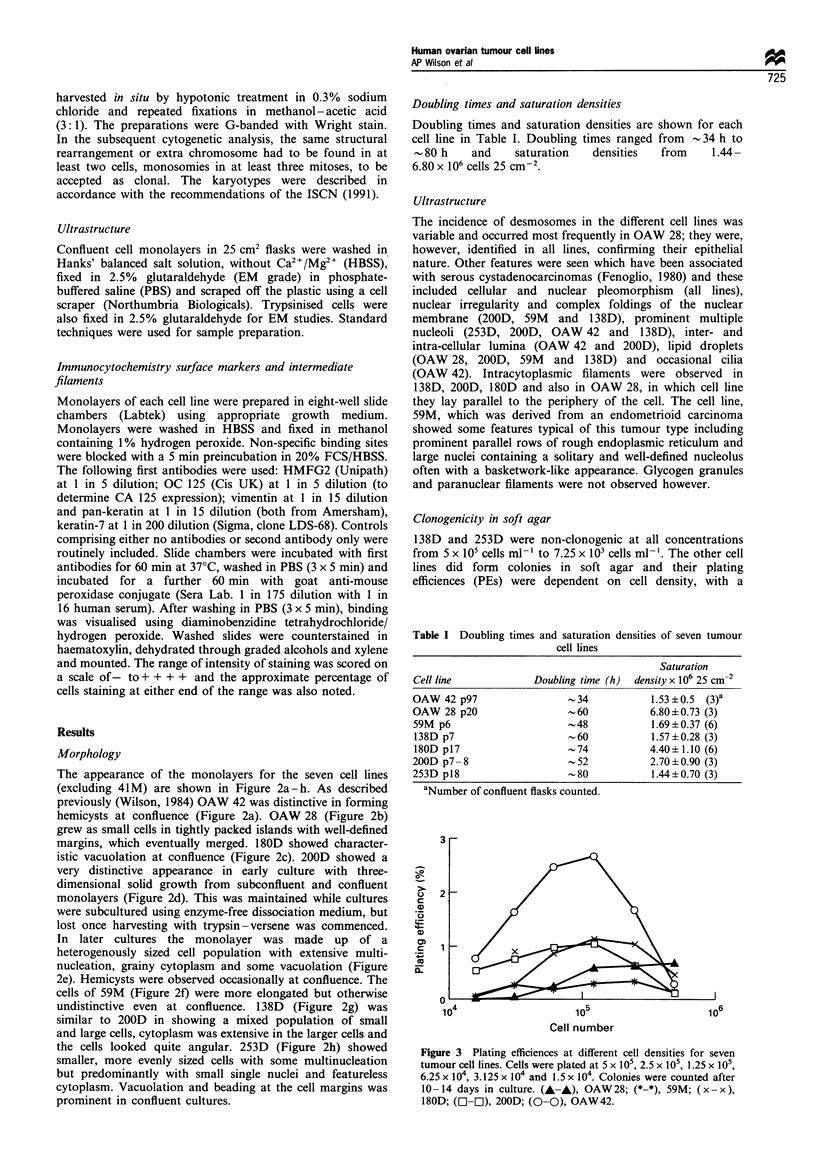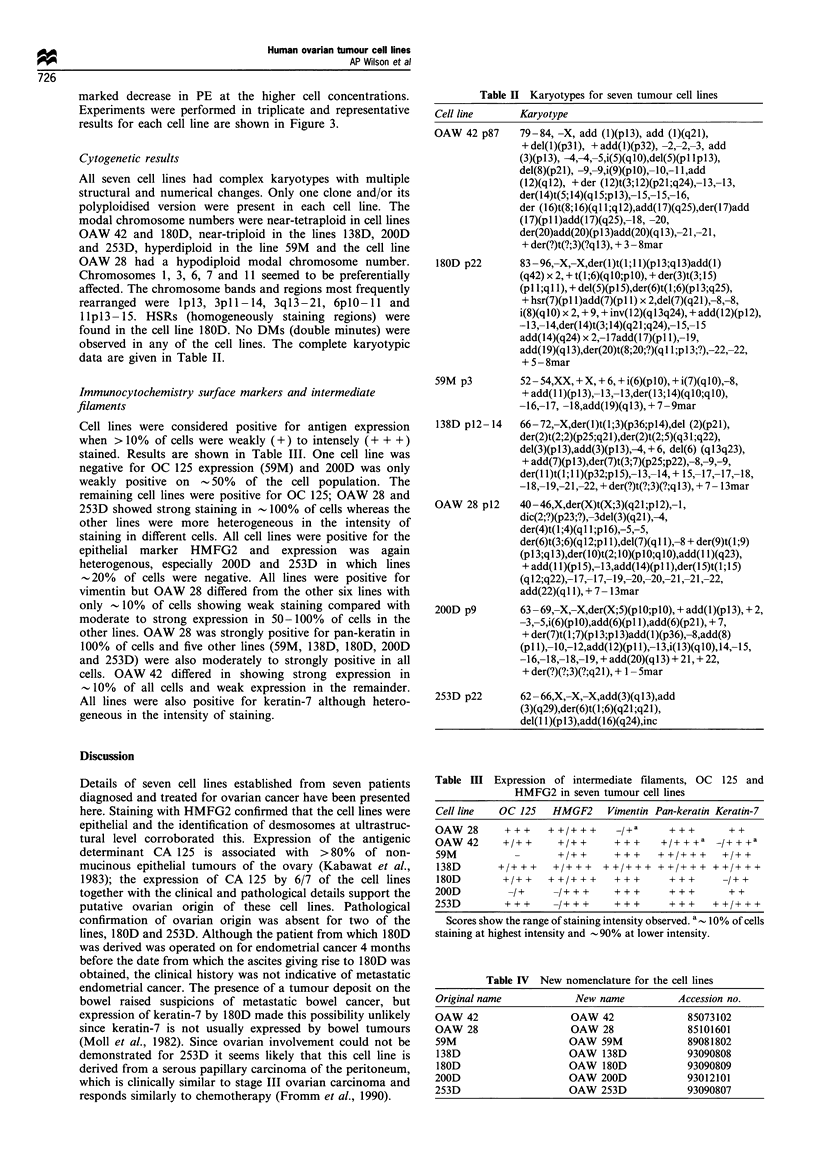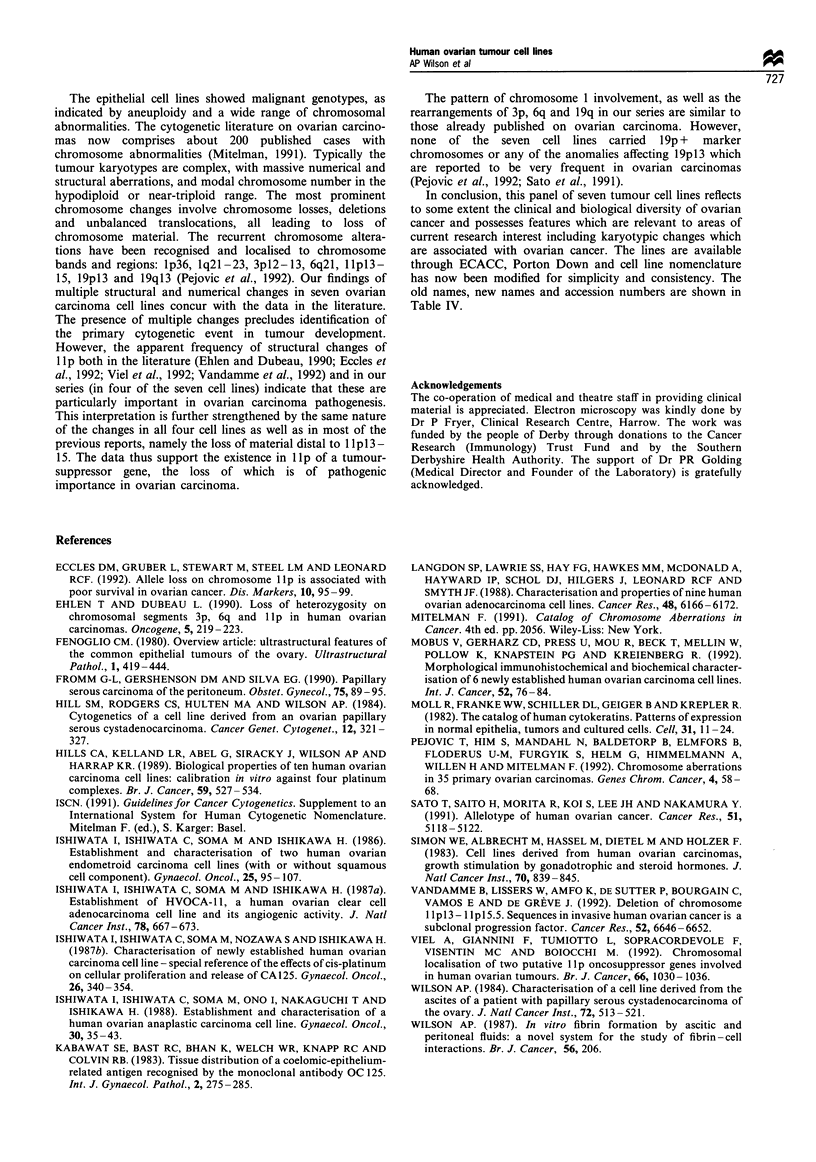# Characterisation of seven human ovarian tumour cell lines.

**DOI:** 10.1038/bjc.1996.428

**Published:** 1996-09

**Authors:** A. P. Wilson, M. Dent, T. Pejovic, L. Hubbold, H. Radford

**Affiliations:** Oncology Research Laboratory, Derby City General Hospital, UK.

## Abstract

**Images:**


British Journal of Cancer (1996) 74, 722-727
© 1996 Stockton Press All rights reserved 0007-0920/96 $12.00

### Characterisation of seven human ovarian tumour cell lines

AP Wilson[1], M Dent[1], T Pejovic[2], L Hubbold[1] and H Radford[1]

[1]*Oncology Research Laboratory, Derby City General Hospital, Uttoxeter Road, Derby DE22 3NE, UK;* [2]*Institute of Oncology, Division of Gynecologic Oncology, University Hospital, S-22185 Lund, Sweden.*

**Summary** Characteristics of a panel of seven human ovarian tumour cell lines are presented. Positive staining with HMFG2 and ultrastructural identification of desmosomes confirmed the epithelial nature of the cell lines. The lines showed wide variations in ploidy, doubling times and clonogenicity in soft agar. Both vimentin and keratin were equally expressed in five lines, one line showed strong preferential expression of keratin and one line showed preferential expression of vimentin. Karyotypic changes associated with ovarian cancer were identified in all the lines. Four of the seven cell lines showed loss of chromosome material distal to 11p13-15. These cell lines offer considerable potential for research into the biology and genetics of ovarian cancer.

**Keywords:** ovarian tumour line; characterisation; heterogeneity; karyotype

In spite of the higher response rates which can be achieved using platinum-based chemotherapy for the treatment of ovarian cancer, 5 year survival rates remain low for advanced disease, and ovarian cancer continues to represent a major clinical challenge. The failure of current treatment regimes to increase survival times reflects the fact that our knowledge of the biology of ovarian cancer has not yet produced information which has had a major impact on therapy and perhaps indicates that improvement in cure rates can only be expected when the biology of the disease is more clearly understood. Ovarian cancer is a heterogeneous disease even within tumours of the same histological type, and a range of lines with a well-documented history and detailed characterisation is therefore useful for exploring this heterogeneity at the cellular level. The literature contains descriptions of ~70 ovarian tumour cell lines, the majority representing reports of only one or two lines. Four groups describe a series containing five or more lines (Simon *et al.*, 1983; Langdon *et al.*, 1988; Mobus *et al.*, 1992; Ishiwata *et al.*, 1986, 1987*a,b* 1988). We present here the main characteristics of seven tumour cell lines derived from patients diagnosed and treated for ovarian cancer. Recent information obtained from DNA fingerprinting and karyotyping has shown that an eighth cell line is identical with one of the cell lines described here (OAW 28 = 41M); this information is documented because OAW 28 and 41M have been distributed previously as two separate cell lines and phenotypic differences have been reported (Hills *et al.*, 1989). One of the cell lines (OAW 42) has been described elsewhere (Wilson, 1984; Hill *et al.*, 1984), and four of them (OAW 42, OAW 28, 41M and 59M) have been used in a panel of ten lines investigated for response to platinum complexes (Hills *et al.*, 1989). Characteristics which are described include morphology, ultrastructure, karyotype and expression of OC125, HMFG2, vimentin, pan-keratin and keratin-7.

### Materials and methods

Seven cell lines developed from ascites (6) and tumour tissue (1) from seven patients with proven or suspected ovarian carcinoma are described here. Patient histories for each line are as follows:

*OAW 42* A detailed history has been described elsewhere (Wilson, 1984). Briefly, the cell line was derived from an ascitic fluid sample of a 46-year-old patient in relapse after a complete response to six courses of 100 mg $m^{-2}$ *cis*-platinum. Histologically, the tumour was a papillary serous cystadenocarcinoma. The patient died 1 month after paracentesis.

*OAW 28* The cell line was derived from the ascites of a 75-year-old patient who had been treated with *cis*-platinum and melphalan, without response. The patient died 3 days after paracentesis. The tumour was described as an adenocarcinoma of the ovary.

*59M* The 65-year-old patient had previously presented with stage 1A carcinoma of the ovary together with *in situ* carcinoma of the endometrium and tubes as separate primaries. After laparotomy she was followed up without further treatment but presented 2 years later with ascites and a lump in the breast which was diagnosed as metastatic ovarian cancer. The cell line was established from the ascitic fluid sample. Following paracentesis, the patient was treated with ifosulphamide and melphalan and although she showed some response, treatment had to be discontinued owing to toxicity. The patient died 10 months after paracentesis. Histologically, the tumour was described as an endometroid carcinoma with clear cell areas.

*138D* The 53-year-old patient had been treated with six courses of carboplatin following laparotomy for bilateral serous cystadenocarcinoma of the ovary, but she relapsed 7 months after the operation. The cell line was established from an ascitic fluid sample taken at relapse. Palliative chemotherapy with melphalan was commenced but the patient died 2 months later.

*180D* The cell line established from the ascites of a 53-year-old patient taken during laparotomy after relapse. Initial diagnosis of carcinoma of the ovary had been made at laparotomy 2 years previously when a bilateral salpingo-oophorectomy and omentectomy was performed but the uterus was left *in situ* because of bulky disease. The patient then received six courses of *cis*-platinum. The uterus was removed 10 months later and found to contain an adenocarcinoma of the endometrium which had invaded but not completely penetrated the myometrium. Further chemotherapy with six courses of carboplatin was then given and another laparotomy was performed on completion. The abdominal cavity was found to contain ascites (180D) and there was a solid tumour deposit on the bowel. Although histologically the tumour was described as a metastatic papillary adenocarcinoma of ovarian origin, the possibility of a bowel primary could not be excluded. The patient died 4 months later.

Correspondence: AP Wilson
Received 5 January 1996; revised 7 March 1996; accepted 12 March 1996

*200D* The cell line was established from the solid tumour of an untreated 51-year-old patient with stage IV disease. The patient died 2 months after laparotomy. Histological examinations of the tumour showed a serous papillary adenocarcinoma with clear cell areas.

*253D* The 74-year-old patient presented initially with an axillary vein thrombosis and investigation revealed the presence of a breast lump. Following mammography, the lump was concluded to be a secondary oedema owing to the thrombosis and breast cancer was excluded. One month later the patient presented with ascites and laparotomy revealed extensive peritoneal disease with involvement of the greater omentum and appendix. The uterus, tubes and ovaries were normal. Histologically, the tumour was described as a serous papillary adenocarcinoma but ovarian origin could not be confirmed. The patient was being treated with warfarin because of the thrombotic episode and cytotoxic treatment was commenced with cyclophosphamide (100 mg day$^{-1}$) and medroxyprogesterone acetate. She presented with ascites 17 months later, from which the cell line was established, and she died 1 month after paracentesis.

### *Establishment of cell lines*

Ascitic fluids were collected without heparin, either at staging laparotomy or by paracentesis. Cells were harvested from the fluids by centrifugation at 1500 r.p.m. for 15 min. Contaminating red blood cells were removed by snap lysis and the cells added to culture flasks in growth medium, PPIGSS (Dulbecco's modification of Eagle medium containing 1 mM pyruvate, penicillin, 1.2 $\mu$g ml$^{-1}$ insulin, 1 mM glutamine, 10% fetal calf serum (FCS) and streptomycin, buffered with 3.7 g l$^{-1}$ of sodium bicarbonate). The solid tumour, from which 200D was established, was very soft and was mechanically disaggregated without enzyme treatment. Cells were resuspended at $\sim 2-5 \times 10^5$ viable cells ml$^{-1}$ and added to plastic tissue culture flasks (Nunc) which were incubated at 37°C in an atmosphere of 95% air/5% carbon dioxide. Stromal cell contamination was a problem with 138D and 180D, and this was eliminated by a combination of differential enzyme treatment and exposure to fibrin. Briefly, monolayers of mixed cell cultures were incubated overnight in a cell-free ascitic fluid known to produce a fibrin mesh on exposure to cells (Wilson, 1987). The mesothelial cells but not the epithelial cells attached to the fibrin, and it was found that a short exposure to trypsin–versene produced complete detachment of the mesothelial cell sheet attached to the fibrin mesh without removing epithelial islands (Figure 1 a and b) which subsequently grew to form a monolayer. OAW 42 and 59M grew well on PPIGSS and quickly developed into continuous cell lines. OAW 28 arose serendipitously from a mixed culture composed mainly of mesothelial cells from two patients (OAW 28 and OAW 53, not reported) and showed rapid expansion from a small population of residual tumour cells. The morphological appearance of these cells was similar to that observed in primary cultures of OAW 28 but not OAW 53, and DNA fingerprinting of the cell line and uncultered banked cells has confirmed that the cell line orginated from patient OAW 28. The line designated 41M grew slowly in primary culture as compact three-dimensional epithelial islands and did not develop into a cell line until the medium was changed to a 50:50 mix of PPIGSS and Ham's F12. Although this line was believed to originate from the ascites of 41M and has been maintained as a separate cell line on P/F12 with 10% FCS, recent information has shown that OAW 28 and 41M have identical karyotypes and DNA fingerprints, indicating cross-contamination of 41M by OAW 28 at the point when culture establishment was believed to have occurred. This information is included here because the line has been made available to a number of investigators and there is evidence of phenotypic differences between OAW 28 and 41M (Hills *et al.*, 1989) possibly as a consequence of different selection pressures owing to continual growth of OAW 28 with PPIGSS medium and 41M with P/F12–10% FCS.

If primary cultures stopped growing while on PPIGSS, they were initially transferred onto PPIGSS/Hams F12 (1:1, v/v) containing 10% FCS. Cell lines 138D, 180D, 200D and 253D all showed deterioration on 10% FCS with gradually enlarging cells without cell division, but further reduction of serum concentration to 5% prevented this change and produced proliferating monolayer cultures. A split ratio of 1:2 was used in the early stages of culture establishment and this was increased to 1:3 or more when appropriate. The use of non-enzymatic dissociation medium (Sigma) for subculture in the early stages of cell line development was found to be beneficial with 200D and 253D, since these cells initially showed poor growth after trypsinisation, and the gentler detachment procedure retained the three-dimensional growth which was characteristic of early passages of these cell lines.

### *Doubling times*

Cells were added at $2 \times 10^4$ ml$^{-1}$ in 10 ml volumes to 25 cm$^2$ flasks and cell numbers determined by harvesting and counting triplicate flasks over a period of $\sim$10 days, using the standard growth medium for each cell line.

**Figure 1** Use of fibrin for separation of epithelial cells and mesothelial cells (original magnification $\times$ 100). (**a**) Before trypsinisation – the fibrin mesh surrounds the epithelial island and mesothelial cells are visible underneath the mesh. (**b**) After trypsinisation – the fibrin mesh is retracting to leave an epithelial island.

*Clonogenicity in soft agar*

Bases of 1 ml of the standard growth medium in 0.5% agar were prepared in 35 mm Petri dishes and cells added as a single cell suspension in a 1 ml overlay containing 0.3% agar. Plates were checked microscopically to confirm that no clumps were present and that agar was solidified. Seven cell concentrations were used, starting at $5 \times 10^5$ ml$^{-1}$ and decreasing in doubling dilutions. After 10–14 days incubation at 37°C in a humidified atmosphere containing 5% carbon dioxide, colonies (>50 $\mu$m) were counted using an inverted microscope at ×100 magnification.

*Cytogenetic methods*

For cytogenetic analysis cells were passed onto glass-chamber slides and cultured for 24–48 h in an appropriate medium. After exposure to colcemid (0.02 $\mu$g ml$^{-1}$), the cultures were

**Figure 2** Morphological appearance of cell lines in culture (original magnification ×115). (**a**) OAW42 with hemicysts forming at confluence. (**b**) OAW28 with islands of small, closely packed cells. (**c**) 180D with vacuolation in confluent cultures. (**d**) 200D with dense, three-dimensional growth. (**e**) 200D with subconfluent culture showing variability in cell size. (**f**) 59M with confluent culture showing small, slightly elongated cells. (**g**) 138D with large, angular cells with small nuclei and extensive cytoplasm. (**h**) 253D with confluent culture showing vacuolation and beading at cell margins.

harvested *in situ* by hypotonic treatment in 0.3% sodium chloride and repeated fixations in methanol–acetic acid (3:1). The preparations were G-banded with Wright stain. In the subsequent cytogenetic analysis, the same structural rearrangement or extra chromosome had to be found in at least two cells, monosomies in at least three mitoses, to be accepted as clonal. The karyotypes were described in accordance with the recommendations of the ISCN (1991).

### *Ultrastructure*

Confluent cell monolayers in 25 $cm^2$ flasks were washed in Hanks' balanced salt solution, without $Ca^{2+}/Mg^{2+}$ (HBSS), fixed in 2.5% glutaraldehyde (EM grade) in phosphate-buffered saline (PBS) and scraped off the plastic using a cell scraper (Northumbria Biologicals). Trypsinised cells were also fixed in 2.5% glutaraldehyde for EM studies. Standard techniques were used for sample preparation.

### *Immunocytochemistry surface markers and intermediate filaments*

Monolayers of each cell line were prepared in eight-well slide chambers (Labtek) using appropriate growth medium. Monolayers were washed in HBSS and fixed in methanol containing 1% hydrogen peroxide. Non-specific binding sites were blocked with a 5 min preincubation in 20% FCS/HBSS. The following first antibodies were used: HMFG2 (Unipath) at 1 in 5 dilution; OC 125 (Cis UK) at 1 in 5 dilution (to determine CA 125 expression); vimentin at 1 in 15 dilution and pan-keratin at 1 in 15 dilution (both from Amersham), keratin-7 at 1 in 200 dilution (Sigma, clone LDS-68). Controls comprising either no antibodies or second antibody only were routinely included. Slide chambers were incubated with first antibodies for 60 min at 37°C, washed in PBS (3 x 5 min) and incubated for a further 60 min with goat anti-mouse peroxidase conjugate (Sera Lab. 1 in 175 dilution with 1 in 16 human serum). After washing in PBS (3 x 5 min), binding was visualised using diaminobenzidine tetrahydrochloride/ hydrogen peroxide. Washed slides were counterstained in haematoxylin, dehydrated through graded alcohols and xylene and mounted. The range of intensity of staining was scored on a scale of – to + + + + and the approximate percentage of cells staining at either end of the range was also noted.

### Results

### *Morphology*

The appearance of the monolayers for the seven cell lines (excluding 41M) are shown in Figure 2a–h. As described previously (Wilson, 1984) OAW 42 was distinctive in forming hemicysts at confluence (Figure 2a). OAW 28 (Figure 2b) grew as small cells in tightly packed islands with well-defined margins, which eventually merged. 180D showed characteristic vacuolation at confluence (Figure 2c). 200D showed a very distinctive appearance in early culture with three-dimensional solid growth from subconfluent and confluent monolayers (Figure 2d). This was maintained while cultures were subcultured using enzyme-free dissociation medium, but lost once harvesting with trypsin–versene was commenced. In later cultures the monolayer was made up of a heterogenously sized cell population with extensive multinucleation, grainy cytoplasm and some vacuolation (Figure 2e). Hemicysts were observed occasionally at confluence. The cells of 59M (Figure 2f) were more elongated but otherwise undistinctive even at confluence. 138D (Figure 2g) was similar to 200D in showing a mixed population of small and large cells, cytoplasm was extensive in the larger cells and the cells looked quite angular. 253D (Figure 2h) showed smaller, more evenly sized cells with some multinucleation but predominantly with small single nuclei and featureless cytoplasm. Vacuolation and beading at the cell margins was prominent in confluent cultures.

### *Doubling times and saturation densities*

Doubling times and saturation densities are shown for each cell line in Table I. Doubling times ranged from ~34 h to ~80 h and saturation densities from 1.44–6.80 x $10^6$ cells 25 $cm^{-2}$.

### *Ultrastructure*

The incidence of desmosomes in the different cell lines was variable and occurred most frequently in OAW 28; they were, however, identified in all lines, confirming their epithelial nature. Other features were seen which have been associated with serous cystadenocarcinomas (Fenoglio, 1980) and these included cellular and nuclear pleomorphism (all lines), nuclear irregularity and complex foldings of the nuclear membrane (200D, 59M and 138D), prominent multiple nucleoli (253D, 200D, OAW 42 and 138D), inter- and intra-cellular lumina (OAW 42 and 200D), lipid droplets (OAW 28, 200D, 59M and 138D) and occasional cilia (OAW 42). Intracytoplasmic filaments were observed in 138D, 200D, 180D and also in OAW 28, in which cell line they lay parallel to the periphery of the cell. The cell line, 59M, which was derived from an endometrioid carcinoma showed some features typical of this tumour type including prominent parallel rows of rough endoplasmic reticulum and large nuclei containing a solitary and well-defined nucleolus often with a basketwork-like appearance. Glycogen granules and paranuclear filaments were not observed however.

### *Clonogenicity in soft agar*

138D and 253D were non-clonogenic at all concentrations from 5 x $10^5$ cells $ml^{-1}$ to 7.25 x $10^3$ cells $ml^{-1}$. The other cell lines did form colonies in soft agar and their plating efficiences (PEs) were dependent on cell density, with a

**Table I** Doubling times and saturation densities of seven tumour cell lines

| *Cell line* | *Doubling time (h)* | *Saturation density* x $10^6$ 25 $cm^{-2}$ |
|---|---|---|
| OAW 42 p97 | ~34 | 1.53 ± 0.5 (3)[a] |
| OAW 28 p20 | ~60 | 6.80 ± 0.73 (3) |
| 59M p6 | ~48 | 1.69 ± 0.37 (6) |
| 138D p7 | ~60 | 1.57 ± 0.28 (3) |
| 180D p17 | ~74 | 4.40 ± 1.10 (6) |
| 200D p7–8 | ~52 | 2.70 ± 0.90 (3) |
| 253D p18 | ~80 | 1.44 ± 0.70 (3) |

[a]Number of confluent flasks counted.

**Figure 3** Plating efficiences at different cell densities for seven tumour cell lines. Cells were plated at 5 x $10^5$, 2.5 x $10^5$, 1.25 x $10^5$, 6.25 x $10^4$, 3.125 x $10^4$ and 1.5 x $10^4$. Colonies were counted after 10–14 days in culture. (▲-▲), OAW28; (*-*), 59M; (x-x), 180D; (□-□), 200D; (O-O), OAW42.

marked decrease in PE at the higher cell concentrations. Experiments were performed in triplicate and representative results for each cell line are shown in Figure 3.

*Cytogenetic results*

All seven cell lines had complex karyotypes with multiple structural and numerical changes. Only one clone and/or its polyploidised version were present in each cell line. The modal chromosome numbers were near-tetraploid in cell lines OAW 42 and 180D, near-triploid in the lines 138D, 200D and 253D, hyperdiploid in the line 59M and the cell line OAW 28 had a hypodiploid modal chromosome number. Chromosomes 1, 3, 6, 7 and 11 seemed to be preferentially affected. The chromosome bands and regions most frequently rearranged were 1p13, 3p11–14, 3q13–21, 6p10–11 and 11p13–15. HSRs (homogeneously staining regions) were found in the cell line 180D. No DMs (double minutes) were observed in any of the cell lines. The complete karyotypic data are given in Table II.

*Immunocytochemistry surface markers and intermediate filaments*

Cell lines were considered positive for antigen expression when >10% of cells were weakly (+) to intensely (+ + +) stained. Results are shown in Table III. One cell line was negative for OC 125 expression (59M) and 200D was only weakly positive on ~50% of the cell population. The remaining cell lines were positive for OC 125; OAW 28 and 253D showed strong staining in ~100% of cells whereas the other lines were more heterogeneous in the intensity of staining in different cells. All cell lines were positive for the epithelial marker HMFG2 and expression was again heterogenous, especially 200D and 253D in which lines ~20% of cells were negative. All lines were positive for vimentin but OAW 28 differed from the other six lines with only ~10% of cells showing weak staining compared with moderate to strong expression in 50–100% of cells in the other lines. OAW 28 was strongly positive for pan-keratin in 100% of cells and five other lines (59M, 138D, 180D, 200D and 253D) were also moderately to strongly positive in all cells. OAW 42 differed in showing strong expression in ~10% of all cells and weak expression in the remainder. All lines were also positive for keratin-7 although heterogeneous in the intensity of staining.

**Table II** Karyotypes for seven tumour cell lines

| *Cell line* | *Karyotype* |
|---|---|
| OAW 42 p87 | 79–84, –X, add (1)(p13), add (1)(q21), + del(1)(p31), + add(1)(p32), –2,–2,–3, add (3)(p13), –4,–4,–5,i(5)(q10),del(5)(p11p13), del(8)(p21), –9,–9,i(9)(p10),–10,–11,add (12)(q12), + der (12)t(3;12)(p21;q24),–13,–13, der(14)t(5;14)(q15;p13),–15,–15,–16, der (16)t(8;16)(q11;q12),add(17)(q25),der(17)add (17)(p11)add(17)(q25),–18, –20, der(20)add(20)(p13)add(20)(q13),–21,–21, + der(?)t(?;3)(?q13), + 3–8mar |
| 180D p22 | 83–96,–X,–X,der(1)t(1;11)(p13;q13)add(1) (q42) × 2, + t(1;6)(q10;p10), + der(3)t(3;15) (p11;q11), + del(5)(p15),der(6)t(1;6)(p13;q25), + hsr(7)(p11)add(7)(p11) × 2,del(7)(q21),–8,–8, i(8)(q10) × 2, + 9, + inv(12)(q13q24), + add(12)(p12), –13,–14,der(14)t(3;14)(q21;q24),–15,–15 add(14)(q24) × 2,–17add(17)(p11),–19, add(19)(q13),der(20)t(8;20;?)(q11;p13;?),–22,–22, + 5–8mar |
| 59M p3 | 52–54,XX, + X, + 6, + i(6)(p10), + i(7)(q10),–8, + add(11)(p13),–13,–13,der(13;14)(q10;q10), –16,–17, –18,add(19)(q13), + 7–9mar |
| 138D p12–14 | 66–72,–X,der(1)t(1;3)(p36;p14),del (2)(p21), der(2)t(2;2)(p25;q21),der(2)t(2;5)(q31;q22), del(3)(p13),add(3)(p13),–4, + 6, del(6) (q13q23), + add(7)(p13),der(7)t(3;7)(p25;p22),–8,–9,–9, der(11)t(1;11)(p32;p15),–13,–14, + 15,–17,–17,–18, –18,–19,–21,–22, + der(?)t(?;3)(?;q13), + 7–13mar |
| OAW 28 p12 | 40–46,X,der(X)t(X;3)(q21;p12),–1, dic(2;?)(p23;?),–3del(3)(q21),–4, der(4)t(1;4)(q11;p16),–5,–5, der(6)t(3;6)(q12;p11),del(7)(q11),–8 + der(9)t(1;9) (p13;q13),der(10)t(2;10)(p10;q10),add(11)(q23), + add(11)(p15),–13,add(14)(p11),der(15)t(1;15) (q12;q22),–17,–17,–19,–20,–20,–21,–21,–22, add(22)(q11), + 7–13mar |
| 200D p9 | 63–69,–X,–X,der(X;5)(p10;p10), + add(1)(p13), + 2, –3,–5,i(6)(p10),add(6)(p11),add(6)(p21), + 7, + der(7)t(1;7)(p13;p13)add(1)(p36),–8,add(8) (p11),–10,–12,add(12)(p11),–13,i(13)(q10),14,–15, –16,–18,–18,–19, + add(20)(q13) + 21, + 22, + der(?)(?;3)(?;q21), + 1–5mar |
| 253D p22 | 62–66,X,–X,–X,add(3)(q13),add (3)(q29),der(6)t(1;6)(q21;q21), del(11)(p13),add(16)(q24),inc |

**Table III** Expression of intermediate filaments, OC 125 and HMFG2 in seven tumour cell lines

| *Cell line* | *OC 125* | *HMGF2* | *Vimentin* | *Pan-keratin* | *Keratin-7* |
|---|---|---|---|---|---|
| OAW 28 | + + + | + +/+ + + | –/+ [a] | + + + | + + |
| OAW 42 | +/+ + | +/+ + | + + + | +/+ + + [a] | –/+ + + [a] |
| 59M | – | +/+ + | + + + | + +/+ + + | +/+ + |
| 138D | +/+ + + | +/+ + + | + +/+ + + | + +/+ + + | + +/+ + + |
| 180D | +/+ + | + +/+ + + | + + + | + + + | –/+ + |
| 200D | –/+ | –/+ + + | + + + | + + + | + + |
| 253D | + + + | –/+ + + | + + + | + + + | + +/+ + + |

Scores show the range of staining intensity observed. [a] ~10% of cells staining at highest intensity and ~90% at lower intensity.

### Discussion

Details of seven cell lines established from seven patients diagnosed and treated for ovarian cancer have been presented here. Staining with HMFG2 confirmed that the cell lines were epithelial and the identification of desmosomes at ultrastructural level corroborated this. Expression of the antigenic determinant CA 125 is associated with >80% of non-mucinous epithelial tumours of the ovary (Kabawat *et al.*, 1983); the expression of CA 125 by 6/7 of the cell lines together with the clinical and pathological details support the putative ovarian origin of these cell lines. Pathological confirmation of ovarian origin was absent for two of the lines, 180D and 253D. Although the patient from which 180D was derived was operated on for endometrial cancer 4 months before the date from which the ascites giving rise to 180D was obtained, the clinical history was not indicative of metastatic endometrial cancer. The presence of a tumour deposit on the bowel raised suspicions of metastatic bowel cancer, but expression of keratin-7 by 180D made this possibility unlikely since keratin-7 is not usually expressed by bowel tumours (Moll *et al.*, 1982). Since ovarian involvement could not be demonstrated for 253D it seems likely that this cell line is derived from a serous papillary carcinoma of the peritoneum, which is clinically similar to stage III ovarian carcinoma and responds similarly to chemotherapy (Fromm *et al.*, 1990).

**Table IV** New nomenclature for the cell lines

| *Original name* | *New name* | *Accession no.* |
|---|---|---|
| OAW 42 | OAW 42 | 85073102 |
| OAW 28 | OAW 28 | 85101601 |
| 59M | OAW 59M | 89081802 |
| 138D | OAW 138D | 93090808 |
| 180D | OAW 180D | 93090809 |
| 200D | OAW 200D | 93012101 |
| 253D | OAW 253D | 93090807 |

The epithelial cell lines showed malignant genotypes, as indicated by aneuploidy and a wide range of chromosomal abnormalities. The cytogenetic literature on ovarian carcinomas now comprises about 200 published cases with chromosome abnormalities (Mitelman, 1991). Typically the tumour karyotypes are complex, with massive numerical and structural aberrations, and modal chromosome number in the hypodiploid or near-triploid range. The most prominent chromosome changes involve chromosome losses, deletions and unbalanced translocations, all leading to loss of chromosome material. The recurrent chromosome alterations have been recognised and localised to chromosome bands and regions: 1p36, 1q21–23, 3p12–13, 6q21, 11p13–15, 19p13 and 19q13 (Pejovic *et al.*, 1992). Our findings of multiple structural and numerical changes in seven ovarian carcinoma cell lines concur with the data in the literature. The presence of multiple changes precludes identification of the primary cytogenetic event in tumour development. However, the apparent frequency of structural changes of 11p both in the literature (Ehlen and Dubeau, 1990; Eccles *et al.*, 1992; Viel *et al.*, 1992; Vandamme *et al.*, 1992) and in our series (in four of the seven cell lines) indicate that these are particularly important in ovarian carcinoma pathogenesis. This interpretation is further strengthened by the same nature of the changes in all four cell lines as well as in most of the previous reports, namely the loss of material distal to 11p13–15. The data thus support the existence in 11p of a tumour-suppressor gene, the loss of which is of pathogenic importance in ovarian carcinoma.

The pattern of chromosome 1 involvement, as well as the rearrangements of 3p, 6q and 19q in our series are similar to those already published on ovarian carcinoma. However, none of the seven cell lines carried 19p+ marker chromosomes or any of the anomalies affecting 19p13 which are reported to be very frequent in ovarian carcinomas (Pejovic *et al.*, 1992; Sato *et al.*, 1991).

In conclusion, this panel of seven tumour cell lines reflects to some extent the clinical and biological diversity of ovarian cancer and possesses features which are relevant to areas of current research interest including karyotypic changes which are associated with ovarian cancer. The lines are available through ECACC, Porton Down and cell line nomenclature has now been modified for simplicity and consistency. The old names, new names and accession numbers are shown in Table IV.

**Acknowledgements**
The co-operation of medical and theatre staff in providing clinical material is appreciated. Electron microscopy was kindly done by Dr P Fryer, Clinical Research Centre, Harrow. The work was funded by the people of Derby through donations to the Cancer Research (Immunology) Trust Fund and by the Southern Derbyshire Health Authority. The support of Dr PR Golding (Medical Director and Founder of the Laboratory) is gratefully acknowledged.

### References

ECCLES DM, GRUBER L, STEWART M, STEEL LM AND LEONARD RCF. (1992). Allele loss on chromosome 11p is associated with poor survival in ovarian cancer. *Dis. Markers*, **10**, 95–99.

EHLEN T AND DUBEAU L. (1990). Loss of heterozygosity on chromosomal segments 3p, 6q and 11p in human ovarian carcinomas. *Oncogene*, **5**, 219–223.

FENOGLIO CM. (1980). Overview article: ultrastructural features of the common epithelial tumours of the ovary. *Ultrastructural Pathol.*, **1**, 419–444.

FROMM G-L, GERSHENSON DM AND SILVA EG. (1990). Papillary serous carcinoma of the peritoneum. *Obstet. Gynecol.*, **75**, 89–95.

HILL SM, RODGERS CS, HULTEN MA AND WILSON AP. (1984). Cytogenetics of a cell line derived from an ovarian papillary serous cystadenocarcinoma. *Cancer Genet. Cytogenet.*, **12**, 321–327.

HILLS CA, KELLAND LR, ABEL G, SIRACKY J, WILSON AP AND HARRAP KR. (1989). Biological properties of ten human ovarian carcinoma cell lines: calibration *in vitro* against four platinum complexes. *Br. J. Cancer*, **59**, 527–534.

ISCN. (1991). *Guidelines for Cancer Cytogenetics*. Supplement to an International System for Human Cytogenetic Nomenclature. Mitelman F. (ed.), S. Karger: Basel.

ISHIWATA I, ISHIWATA C, SOMA M AND ISHIKAWA H. (1986). Establishment and characterisation of two human ovarian endometroid carcinoma cell lines (with or without squamous cell component). *Gynaecol. Oncol.*, **25**, 95–107.

ISHIWATA I, ISHIWATA C, SOMA M AND ISHIKAWA H. (1987*a*). Establishment of HVOCA-11, a human ovarian clear cell adenocarcinoma cell line and its angiogenic activity. *J. Natl Cancer Inst.*, **78**, 667–673.

ISHIWATA I, ISHIWATA C, SOMA M, NOZAWA S AND ISHIKAWA H. (1987*b*). Characterisation of newly established human ovarian carcinoma cell line – special reference of the effects of cis-platinum on cellular proliferation and release of CA125. *Gynaecol. Oncol.*, **26**, 340–354.

ISHIWATA I, ISHIWATA C, SOMA M, ONO I, NAKAGUCHI T AND ISHIKAWA H. (1988). Establishment and characterisation of a human ovarian anaplastic carcinoma cell line. *Gynaecol. Oncol.*, **30**, 35–43.

KABAWAT SE, BAST RC, BHAN K, WELCH WR, KNAPP RC AND COLVIN RB. (1983). Tissue distribution of a coelomic-epithelium-related antigen recognised by the monoclonal antibody OC125. *Int. J. Gynaecol. Pathol.*, **2**, 275–285.

LANGDON SP, LAWRIE SS, HAY FG, HAWKES MM, MCDONALD A, HAYWARD IP, SCHOL DJ, HILGERS J, LEONARD RCF AND SMYTH JF. (1988). Characterisation and properties of nine human ovarian adenocarcinoma cell lines. *Cancer Res.*, **48**, 6166–6172.

MITELMAN F. (1991). *Catalog of Chromosome Aberrations in Cancer*. 4th ed. pp. 2056. Wiley-Liss: New York.

MOBUS V, GERHARZ CD, PRESS U, MOU R, BECK T, MELLIN W, POLLOW K, KNAPSTEIN PG AND KREIENBERG R. (1992). Morphological immunohistochemical and biochemical characterisation of 6 newly established human ovarian carcinoma cell lines. *Int. J. Cancer*, **52**, 76–84.

MOLL R, FRANKE WW, SCHILLER DL, GEIGER B AND KREPLER R. (1982). The catalog of human cytokeratins. Patterns of expression in normal epithelia, tumors and cultured cells. *Cell*, **31**, 11–24.

PEJOVIC T, HIM S, MANDAHL N, BALDETORP B, ELMFORS B, FLODERUS U-M, FURGYIK S, HELM G, HIMMELMANN A, WILLEN H AND MITELMAN F. (1992). Chromosome aberrations in 35 primary ovarian carcinomas. *Genes Chrom. Cancer*, **4**, 58–68.

SATO T, SAITO H, MORITA R, KOI S, LEE JH AND NAKAMURA Y. (1991). Allelotype of human ovarian cancer. *Cancer Res.*, **51**, 5118–5122.

SIMON WE, ALBRECHT M, HASSEL M, DIETEL M AND HOLZER F. (1983). Cell lines derived from human ovarian carcinomas, growth stimulation by gonadotrophic and steroid hormones. *J. Natl Cancer Inst.*, **70**, 839–845.

VANDAMME B, LISSERS W, AMFO K, DE SUTTER P, BOURGAIN C, VAMOS E AND DE GRÈVE J. (1992). Deletion of chromosome 11p13–11p15.5. Sequences in invasive human ovarian cancer is a subclonal progression factor. *Cancer Res.*, **52**, 6646–6652.

VIEL A, GIANNINI F, TUMIOTTO L, SOPRACORDEVOLE F, VISENTIN MC AND BOIOCCHI M. (1992). Chromosomal localisation of two putative 11p oncosuppressor genes involved in human ovarian tumours. *Br. J. Cancer*, **66**, 1030–1036.

WILSON AP. (1984). Characterisation of a cell line derived from the ascites of a patient with papillary serous cystadenocarcinoma of the ovary. *J. Natl Cancer Inst.*, **72**, 513–521.

WILSON AP. (1987). *In vitro* fibrin formation by ascitic and peritoneal fluids: a novel system for the study of fibrin–cell interactions. *Br. J. Cancer*, **56**, 206.